# The Interaction between Exercise and Marital Status on Depression: A Cross-Sectional Study of the Taiwan Biobank

**DOI:** 10.3390/ijerph19031876

**Published:** 2022-02-08

**Authors:** Ming-Yi Hsu, Shih-Chien Huang, Pang-Li Liu, Kwok-Tak Yeung, Yu-Ming Wang, Hao-Jan Yang

**Affiliations:** 1 Department of Nursing, Chung Shan Medical University, Taichung 40201, Taiwan; mingyi@csmu.edu.tw; 2 Department of Nursing, Chung Shan Medical University Hospital, Taichung 40201, Taiwan; 3 Department of Nutrition, Chung Shan Medical University, Taichung 40201, Taiwan; schuang@csmu.edu.tw; 4 Department of Health Industry Technology Management, Chung Shan Medical University, Taichung 40201, Taiwan; 5 Department of Nutrition, Chung Shan Medical University Hospital, Taichung 40201, Taiwan; 6 Department of Health Promotion, Taiwan Adventist College, Yu Chih 555, Taiwan; lukeplliu@sdatac.ogr.tw; 7 Department of Occupational Therapy, Chung Shan Medical University, Taichung 40201, Taiwan; gordon963@gmail.com; 8 Occupational Therapy Room, Chung Shan Medical University Hospital, Taichung 40201, Taiwan; 9 Department of Psychology, Chung Shan Medical University, Taichung 40201, Taiwan; 10 Clinical Psychological Room, Chung Shan Medical University Hospital, Taichung 40201, Taiwan; 11 Department of Public Health, College of Health Care and Management, Chung Shan Medical University, Taichung 40201, Taiwan; 12 Department of Family and Community Medicine, Chung Shan Medical University Hospital, Taichung 40201, Taiwan

**Keywords:** marital status, depression, exercise, PHQ-2, gender

## Abstract

Few studies evaluating the relationship between depression and exercise consider peoples’ socio-demographic characteristics. This cross-sectional study investigated the interaction between exercise and marital status and depression in Taiwanese adults. Data from the 2-item Patient Health Questionnaire (PHQ-2) was recruited from the Taiwan Biobank. Participants indicated their exercise status, showing 5015 no-exercise cases and 3407 exercise cases. Marital status, including unmarried, divorced or separated, and widowed, were all significant, especially among the no-exercise group. The relationship between exercise/no exercise and marital status was examined; no exercise and unmarried, divorced or separated, and widowed, as well as exercise and married were significant to PHQ-2. Gender was significant in both the married and unmarried groups. The association between exercise, marital status, gender, and education on PHQ-2 score was also significant. Married people, especially men, had lower depression scores. Additionally, exercise had a protective effect against depression for unmarried people, especially women.

## 1. Introduction

Depression is an illness that can affect anyone of any gender, at any age or life stage. There are many negative consequences, both immediate and long-term, associated with mental disorders, including the impairment of social functioning, poor education and employment attainment and achievement, and increased risks of self-harm and suicide [1,2]. The occurrence of depressive symptoms at low, mild, and moderate-to-severe levels was shown by a study to be 55.5%, 23.0%, 19.0%, and 13.5%, respectively [3]. A recent study revealed that the prevalence of depression symptoms was 48.2% among Chinese adolescents during the pandemic, although moderate and highly active physical activity was associated with lower levels of depression symptoms [4]. Research also shows that more than half of all people exhibiting symptoms of depression do not receive effective treatment, such as antidepressant medication. Possible reasons for this include misdiagnosis, the perceived stigma around mental health, and a lack of access to resources. Oexle et al. (2017) studied more than 200 individuals with mental illness over a period of two years, and found that stigma and discrimination can contribute to worsening symptoms and a reduced likelihood of receiving treatment, with greater self-stigma being associated with poorer recovery from mental illness after one and two years [5]. Available evidence suggests not only that physical exercise has clear health benefits and can be applied to depressive disorders [6], but also that it might be comparable to antidepressant treatment for depressed patients [7]. Many studies show that exercise is linked to a lower mental health burden. While exercise can be just as effective as antidepressants in less severe cases, evidence of exercise alone as a generally effective treatment for depression is still inconclusive [8].

Research has shown that increasing age, gender, residence, marital status, presence of co-morbidities, visual impairment, previous falls, loneliness, and fear of falling were significant determinants for developing depressive symptoms [1,7]. Research into the interaction between depression and exercise that focused on the socio-demographic characteristics of gender showed that women were at higher risk for increased depression symptoms, and that they reported a higher level of depression than men [9]. Marital status is another socio-demographic characteristic that might indirectly affect mental health. While some studies have shown that after getting married people become less active [10,11], other studies have shown opposite results [12,13]. Chekroud et al. (2018), using the genetic data of 300,000 adults, found that exercise had a protective effect against depression. The study showed that people with higher levels of physical activity had fewer occurrences of major depressive disorders, and that exercising for 45 min for three to five times a week was associated with the greatest benefits [4].

Although the preventive effect of exercise on depression can be seen from the above studies, a review of the literature revealed a lack of research focusing directly on the interaction between depression and exercise considering the socio-demographic characteristic of marital status. This study aimed to examine: (1) whether individuals who exercise are less depressed than individuals who do not; and (2) whether exercise interacts with marital status on the occurrence of depression. Gender difference was also addressed in this interaction.

## 2. Materials and Methods

### 2.1. Study Population

In this cross-sectional study, data of 8422 individuals were collected from the Taiwan Biobank. The sample size was determined according to the suggestion proposed by Charan and Biswas (2013) in this cross-sectional study with the dichotomous exposure variables (exercise and marital status) and continuous outcome (depression score) [14]. With an expected mean difference (d) of 2.06 on depression symptoms between the exercise and no-exercise group (Gourgouvelis et al., 2018), a total sample size of 6412 was sufficient for statistical power [15]. Considering an attrition rate of 15%, we therefore enrolled 8422 participants in this study.

The Taiwan Biobank was created to collect and store biological and lifestyle information as a resource that researchers might use to determine health risk factors in Taiwan. Recruitment information about enrolment in the Taiwan Biobank project is available to the public through media, posters, brochures, and websites. The data in the Biobank are obtained through 29 centers throughout Taiwan for recruiting participants; each city or county has at least one center. After interested volunteers sign the consent form, researchers interview them and fill in questionnaire items including age, sex, and lifestyle (e.g., cigarette smoking, exercise, and alcohol drinking). Anthropometric data (e.g., weight and height), and other health data are determined by physical examinations. The Taiwan Biobank complies with relevant data protection and privacy regulations. The questionnaire used to collect the date included the PHQ-2 questions, as well as a number of demographic items: exercise (no/yes), marital status (married; unmarried; divorced or separated; widowed), gender (female/male), age, education level (university and above; senior high school; junior high school; elementary school), live alone (no/yes), body mass index (normal; underweight; overweight; obese), smoking habits (never; former; current smoker), and drinking habits (no; former; current). Exercise was defined as regular exercise: 3 times a week and over 30 min each time. The four levels of BMI were defined as underweight (under 18.5 kg/m^2^), normal weight (18.5 to 25), overweight (25 to 30), and obese (over 30). Never smoking was defined as never having smoked or having smoked for less than 6 months. Former smoking was defined as having smoked for more than 6 months but seldom or never smoking now. Current smoking was defined as having smoked for more than 6 months and currently smoking. Non-drinkers were defined as having no history of drinking alcohol or weekly drinking of less than 150 cc of alcohol for 6 consecutive months; former drinkers were defined as having abstained from drinking for over 6 months. Current drinkers were defined as drinking at least 150 cc of alcohol weekly for 6 consecutive months. The study was approved by the Institutional Review Board of Chung Shan Medical University Hospital (CS1-20009), and all subjects consented prior to participation.

### 2.2. Questionnaire

The Patient Health Questionnaire (PHQ) was first developed to assess generalized anxiety disorder. The questionnaire used in this study included the 2-item Patient Health Questionnaire (PHQ-2) [16,17]. Adapted from the PHQ-9, this tool has been used as a first step in depression screening to identify individuals who require additional evaluation [17]. The PHQ-2 has been shown to have excellent operating characteristics for assessing depressive disorders [18]. The PHQ-2 begins with the stem question: “Over the last 2 weeks, how often have you been bothered by the following problems?” Response options are “not at all”, “several days”, “more than half the days”, and “nearly every day”, scored as 0, 1, 2, and 3, respectively. We used the two questions contained in the PHQ-2, yielding total PHQ-2 scores ranging from 0 to 6.

### 2.3. Statistical Analysis

We used the chi-square test to compare categorical data. The association between categorical and continuous variables was determined using the Student’s *t*-test. Demographic data for exercise cases and non-exercise cases were compared using Student’s *t*-test (for continuous variables) and chi-square test (for categorical variables). To evaluate the association of exercise with PHQ-2, we examined the correlations through linear regression analysis to evaluate the β and their *p*-values, adjusting for covariates. Multiple linear regression of PHQ-2 on exercise was performed by stratifying marital status and adjusting for age, education level, BMI, alcohol drinking, and cigarette smoking habits. In addition, the interaction between marital status and exercise was put into a multiple linear regression model to examine their moderating effect on depression. Data management and statistical analyses were performed using SAS 9.3 software (SAS Institute, Cary, NC, USA).

## 3. Results

Table 1 shows the demographic characteristics of the 5015 no-exercise cases and 3407 exercise cases. The demographic characteristics (Table 1) show that the study group included more married and exercise cases (about 70%) than unmarried and exercise cases.

Table 2 shows the multiple linear regression of PHQ-2. Smoking habits were significant to PHQ-2, including both former smoking (β = 0.08215, *p*-value = 0.0263) and current smoking (β = 0.17853, *p*-value < 0.0001). Exercise and education levels were also significant to PHQ-2. The β were −0.12410 (*p*-value < 0.0001) for the exercise group. Marital status, including unmarried (β = 0.19605, *p*-value < 0.0001), divorced or separated, and widowed, were all significant to PHQ-2. Moreover, the interaction between exercise and marital status was significant to PHQ-2.

Multiple linear regression of PHQ-2, stratified by exercise, is presented in Table 3. Marital status—including unmarried (β = 0.2144, *p*-value < 0.0001), divorced or separated (β = 0.15503, *p*-value = 0.0065), and widowed (β = 0.32346, *p*-value < 0.0001)—was significant in the no-exercise group. Interactions between exercise, gender, and marital status were found to be significant to PHQ-2. While there was no significant difference between exercising men and women, both of these groups indicated lower depression levels than no-exercise females. Regardless of whether they exercised or not, married cases indicate lower depression levels than cases of other marital statuses. Unmarried cases who exercised had lower depression levels than unmarried cases who did not exercise (β = 0.2144 vs. 0.14872).

Table 4 presents the multiple linear regression of PHQ-2, stratifying by marital status. Exercise was significant in married (β = −0.09550, *p*-value = 0.0001), unmarried (β = −0.20620, *p*-value = 0.0124), and widowed groups (β = −0.38743, *p*-value = 0.0003). Gender was also significant in married (β = −0.12031, *p*-value < 0.0001), and divorced or separated groups (β = −0.30605, *p*-value = 0.0119).

Multiple linear regression of PHQ-2 to marital status and exercise are shown in Table 5. In the relationship of marital status to exercise, no exercise and unmarried (β = 0.22849, *p*-value < 0.0001), no exercise and divorced or separated (β = 0.16138, *p*-value = 0.0019), no exercise and widowed (β = 0.31890, *p*-value < 0.0001), and exercise and married (β = −0.09160, *p*-value = 0.0005) were significant to PHQ-2.

## 4. Discussion

Depression has a great impact on quality of life. An emerging paradigm in the treatment of depression is lifestyle medicine. However, while modification of lifestyle risk factors has the potential to prevent and treat depression, few studies have been conducted into the relationship between depression and exercise (or physical activity, PA) considering peoples’ socio-demographic characteristics. In this study, we found that there is an interaction between exercise and marital status, and the risk of developing depression for both men and women. Regardless of marital status, individuals who exercised were more likely to have lower depressive scores than those who did not exercise, and for married individuals who exercised the reduction in risk of depression was significant.

The Patient Health Questionnaire–2 (PHQ-2) can be used as a first step to screening for depression [17]. Taylor et al. (2014), through an analysis of self-reported data from a large international sample studying lifestyle factors, examined depression risk using the Patient Health Questionnaire-2 (PHQ-2) [16]. Approximately one-fifth (19.3%) of their sample screened positive for depression (PHQ-2 score ≥ 3). Regression analyses showed that marked social isolation, taking interferon, obesity, smoking, and low levels of exercise were associated with greater depression risk. Another 2018 study used PHQ-2 on 116 spinal cord injury/dysfunction (SCI/D) outpatients at Veterans Affairs Medical Center, and found that PHQ-2 showed promise as a clinically useful screener in the community-residing SCI/D population [19]. Our study used PHQ-2 to examine depression using the data of 8422 subjects taken from the Taiwan Biobank, and we also found both a significant relationship between smoking history and PHQ-2 and a clear indication that exercise, when examined with marital status, was significant to PHQ-2. However, our study found no significant relationship between BMI (including obesity) and PHQ-2.

Studies in the field of physiology explain the mental health benefits accrued from regular exercise. In Brown, Heesch, and Miller, (2009) neuroscientists noticed that patients suffering from depression had a smaller hippocampus [11]. They further stated that exercise supports nerve cell growth in this region of the brain, improving nerve cell connections, which helps relieve depression. Therefore, an improvement in brain function makes us feel better, which helps relieve depression. In another study, a lower mental health burden was found to be associated with all exercise types of a duration of 45 min and frequency of three to five times per week [20]. The authors of this study examined a variety of exercise types of durations of 30, 60, and 90 min, and also concluded that duration of 45 min was best for lowering mental health burden. However, our study sample consisted of adults whose culture and exercise types differed from the subjects in previous research. Our research findings showed that those who exercised regularly—three times a week and over 30 min each time—were less likely to be depressed than those who did not exercise. In today’s fast-paced society, it is good news that clear benefits can be obtained with even a half-hour’s investment.

When people enter a partner relationship, depression is significantly associated with subsequently lower relationship support and higher conflict. [21]. Nomaguchi and Bianchi (2004) found that entering into a partner relationship also appears to influence exercise habits. Their study found that men spend more time on exercise than women and that, although married men spend less time on exercise than unmarried men, they still spend more time on exercise than women [22]. Another study also shows that while men exercise for enjoyment, women exercise for weight loss and toning [23]. The Nomaguchi and Bianchi (2004) study also indicated that among American men and women aged 18 to 64 (*N* = 13,496), married adults spend less time exercising than unmarried adults [22]. Several other studies have also found that participation rates in physical activities are lower for married adults compared with unmarried adults [24,25]. This might be because married people often participate in more social activities such as family gatherings, or activities easily combined with housework and childcare, and spend less time on the type of activities they enjoy during a moment of freedom away from work and family, activities that Nomaguchi and Bianchi (2004) considered, along with exercise, as individual leisure pursuits [22]. In more recent research, Cavazzotto et al. (2022) studied 561,837 individuals from 18 to 64 years of age in order to identify the age and sex-related associations between marital status and PA [26]. When compared to single individuals, younger married participants were less likely to do more than 150 min of PA/week. However, after the age of 40, married women were more likely to complete more than 150 min of PA/week than single women. There were no differences among married men by age group.

In our study, the average ages for the no exercise and exercise groups were 47.01 (SD ± 0.15) and 54.54 (SD ± 0.17), respectively, and we found that no matter whether they exercised or not, married participants still had lower depression levels than participants of other marital statuses, and this was especially true for married men. Jeong et al. (2009) indicated that when Asian married women take on the responsibilities of a wife, they also take on the additional responsibilities of a daughter-in-law, which often brings the additional burden of living with parents-in-law, older relatives, and step/children [27]. Asian married women carry a greater burden in childrearing and often face greater difficulties adjusting to married life, communicating with their husbands, and finding time for exercise, and these could pose the risk of emotional distress or depression [28,29]. A significant relationship has been shown between depressive symptoms and family stress and conflict [30], and that unstable family relationships often pre-exist depressive symptoms [31]. Bulloch et al. (2017) studied the relationship between depression and marital status and found that the odds ratios of depression were smaller for females (vs. males) who were single, widowed or separated, compared to married people [32]. Findings from our research indicated that married cases had lower levels of depression than those of other marital statuses. Furthermore, we found married men had lower depression scores than men or women in other marital statuses. An even more important finding from our research was that differences in depression levels became non-significant between women and men when women exercised, especially single women.

Marques et al. (2019) found that whether single or married, engaging in leisure-time physical activity (LTPA) was negatively and linearly related to depression symptoms, independently of being men or women, and that, in a marriage relationship, married women had lower depression levels than married men [33]. Looking only at older married couples, Monin et al. (2015) examined whether each spouse’s physical activity predicted changes in their own and their partner’s depressive symptoms. Their results showed that the husbands’ physical activity significantly predicted a decrease in their own depressive symptoms and also predicted their wives’ physical activity and depressive symptoms. They also found that the odds ratios of depression were smaller for females (vs. males) who were single, widowed or separated compared to married people [34]. While there is no conclusion with regard to the relationship between exercise, gender and marital status, important findings from our research show that exercise has a protective effect against depression for unmarried cases—especially unmarried and divorced women. We also learned from our study that widows who exercise are much less depressed than widows who do not exercise. Exercise is apparently a protective factor for women.

In summary, a significant effect of the interaction between exercise and marital status on depression (as measured by PHQ-2) was found in our study. The results of this study will provide a foundation for further study on the effects that the limited opportunities for leisure activities occasioned by the COVID-19 outbreak might have on mental health, and help identify whether different marital status serves as a protective factor during such stressful times. Furthermore, the results of the present study have implications for policy makers, as they demonstrate the value of promoting exercise as an effective public mental health measure. As our findings highlight the importance of exercise interventions for married, single, or divorced women due to its protective effect, the study may also be relevant for researchers assessing the relationship between exercise and depression in marital relationships. Finally, this study is limited by the fact that, due to the unavailability of relevant data concerning the type, intensity, or frequency of exercise in the Taiwan Biobank dataset, qualitative rather than quantitative data was used to assess variables regarding exercise. This is a cross-sectional study, and is thus unable to determine the time course of the causal relationships between the variables. This study is also limited by its reliance on volunteer participants, which may create problems related to representation and generalization. Future studies could focus on those cases whose diagnosis was confirmed by a medical doctor.

## 5. Conclusions

The association between PHQ-2 score and exercise, marital status, gender, and education was found to be significant. Married people (regardless of whether they exercise), especially men, had lower depression scores than people of other marital statuses. Among unmarried people, those who exercised suffered lower levels depression than those who did not. Additionally, exercise had a protective effect against depression for unmarried people, especially women. Therefore, it is clear that, for all segments of the population, regardless of gender and marital status, regular exercise can contribute to lower levels of depression and a higher quality of life.

## Figures and Tables

**Table 1:** Demographic characteristics of PHQ-2.

Variables	No Exercise (*n* = 5015)	Exercise (*n* = 3407)	*p*-Value
Marital status			<0.0001
Married	3643 (72.64)	2674 (78.49)	
Unmarried	773 (15.41)	299 (8.78)	
Divorced or Separated	410 (8.18)	227 (6.66)	
Widowed	189 (3.77)	207 (6.08)	
Gender			0.6928
Female	3024 (60.30)	2069 (60.73)	
Male	1991 (39.70)	1338 (39.27)	
Age	47.01 ± 0.15	54.54 ± 0.17	<0.0001
Education level			<0.0001
University and above	2669 (53.22)	1537 (45.11)	
Senior high school	1378 (27.48)	984 (28.88)	
Junior high school	334 (6.66)	310 (9.10)	
Elementary and below	634 (12.64)	576 (16.91)	
Live alone			0.9839
No	4627 (92.26)	3143 (92.25)	
Yes	388 (7.74)	264 (7.75)	
BMI			<0.0001
Normal	2353 (46.92)	1734 (50.90)	
Underweight	165 (3.29)	82 (2.41)	
Overweight	1426 (28.43)	1009 (29.62)	
Obese	1071 (21.36)	582 (17.08)	
Smoking habit			<0.0001
No	3936 (78.48)	2735 (80.28)	
Former	506 (10.09)	470 (13.80)	
Current	573 (11.43)	202 (5.93)	
Drinking habit			<0.0001
No	4551 (90.75)	3043 (89.32)	
Former	119 (2.37)	144 (4.23)	
Current	345 (6.88)	220 (6.46)	

BMI, body mass index; PHQ-2, Patient Health Questionnaire-2.

**Table 2:** Multiple linear regression of PHQ-2(34).

Variables	β	*p*-Value
Exercise (ref: No)		
Yes	−0.12410	<0.0001
Marital status (ref: Married)		
Unmarried	0.19605	<0.0001
Divorced or separation	0.13135	0.0020
Widowed	0.19207	0.0003
Gender (ref: Female)		
Male	−0.11402	<0.0001
Age	−0.01702	<0.0001
Education level (ref: University and above)		
Senior high school	−0.05919	0.0231
Junior high school	−0.08177	0.0572
Elementary and below	−0.08451	0.0136
Live alone (ref: No)		
Yes	0.07269	0.0954
BMI (ref: Normal)		
Underweight	−0.06521	0.3067
Overweight	−0.00187	0.9415
Obese	−0.01360	0.6386
Smoking habit (ref: No)		
Former	0.08215	0.0263
Current	0.17853	<0.0001
Drinking habit (ref: No)		
Former	0.09433	0.1343
Current	−0.00817	0.8546
Interaction	0.0022	

BMI, body mass index; PHQ-2, Patient Health Questionnaire-2.

**Table 3:** Multiple linear regression of PHQ-2 stratified by exercise.

Variables	No Exercise		Exercise	
	β	*p*-Value	β	*p*-Value
Marital status				
Unmarried	0.2144	<0.0001	0.14872	0.0111
Divorced or separation	0.15503	0.0065	0.09622	0.1238
Widowed	0.32346	<0.0001	0.06861	0.2891
Gender (ref: Female)				
Male	−0.14153	<0.0001	−0.06390	0.0807
Age:	−0.01763	<0.0001	−0.01645	<0.0001
Education level (ref: University and above)				
Senior high school	−0.08948	0.0150	−0.01247	0.7245
Junior high school	−0.08691	0.1739	−0.06486	0.2355
Elementary and below	−0.14400	0.0044	−0.01075	0.8066
Live alone (ref: No)				
Yes	0.09239	0.1257	0.05470	0.3708
BMI (ref: Normal)				
Underweight	−0.04280	0.6140	−0.12196	0.2008
Overweight	−0.00298	0.9344	0.00077575	0.9818
Obese	0.01955	0.6226	−0.06387	0.1206
Smoking habit (ref: No)				
Former	0.09496	0.0775	0.05515	0.2549
Current	0.20293	0.0001	0.13520	0.0435
Drinking habit(ref: No)				
Former	0.14059	0.1611	0.05327	0.4765
Current	0.00598	0.9227	−0.02612	0.6753

BMI, body mass index; PHQ-2, Patient Health Questionnaire-2.

**Table 4:** Multiple linear regression of PHQ-2, stratifying by marital status.

Variables	Married		Unmarried		Divorced or Separation		Widowed	
	β	*p*-Value	β	*p*-Value	β	*p*-Value	β	*p*-Value
Exercise								
Yes	−0.09550	0.0001	−0.20620	0.0124	−0.14732	0.1218	−0.38743	0.0003
Gender (ref: Female)								
Male	−0.12031	<0.0001	0.00926	0.9126	−0.30605	0.0119	−0.21875	0.2926
Age	−0.01628	<0.0001	−0.02202	<0.0001	−0.01803	0.0006	−0.01409	0.0582
Education level (ref: University and above)								
Senior high school	−0.07189	0.0098	−0.08755	0.4033	0.05530	0.5889	−0.14116	0.3377
Junior high school	−0.10284	0.0257	−0.26287	0.3385	0.01762	0.9120	−0.09010	0.5829
Elementary and below	−0.08475	0.0200	−0.14505	0.4299	−0.08025	0.5624	−0.14902	0.3054
Live alone (ref: No)								
Yes	0.17722	0.0741	0.05180	0.5398	0.11983	0.2241	0.05304	0.6543
BMI (ref: Normal)								
Underweight	−0.17516	0.0161	0.11581	0.4635	0.45048	0.2229	0.41660	0.2775
Overweight	0.00607	0.8257	0.03756	0.6904	−0.06269	0.5405	−0.11694	0.3151
Obese	0.01418	0.6527	−0.11952	0.2268	0.11805	0.3238	−0.40455	0.0050
Smoking habit (ref: No)								
Former	0.03897	0.3170	0.25555	0.0901	0.37233	0.0156	0.16473	0.5004
Current	0.16215	0.0004	0.17972	0.1940	0.32274	0.0210	0.41738	0.1286
Drinking habit (ref: No)								
Former	0.02321	0.7363	0.32742	0.1483	0.28840	0.1975	0.26085	0.4378
Current	−0.00886	0.8512	0.05951	0.7286	−0.00922	0.9584	−0.19494	0.5224

BMI, body mass index; PHQ-2, Patient Health Questionnaire-2.

**Table 5:** Multiple linear regression of PHQ-2 to marital status and exercise.

Variables	β	*p*-Value
Exercise, marital status (ref: No exercise, Married)		
No exercise, Unmarried	0.22849	<0.0001
No exercise, Divorced or separated	0.16138	0.0019
No exercise, Widowed	0.31890	<0.0001
Exercise, Married	−0.09160	0.0005
Exercise, Unmarried	0.02044	0.7330
Exercise, Divorced or separated	−0.01283	0.8515
Exercise, Widowed	−0.01939	0.7888
Gender (ref: Female)		
Male	−0.11210	<0.0001
Age	−0.01729	<0.0001
Education level (ref: University and above)		
Senior high school	−0.05937	0.0226
Junior high school	−0.08239	0.0552
Elementary and below	−0.08364	0.0146
Live alone (ref: No)		
Yes	0.07947	0.0692
BMI (ref: Normal)		
Underweight	−0.06920	0.2780
Overweight	−0.00234	0.9266
Obese	−0.01453	0.6160
Smoking habit (ref: No)		
Former	0.07940	0.0318
Current	0.17952	<0.0001
Drinking habit (ref: No)		
Former	0.09422	0.1347
Current	−0.01049	0.8139

BMI, body mass index; PHQ-2, Patient Health Questionnaire-2.

## Data Availability

The datasets used during the current study are available from the corresponding author on reasonable request.
